# Persistence of Different Forms of Transient RNAi during Apoptosis in Mammalian Cells

**DOI:** 10.1371/journal.pone.0012263

**Published:** 2010-08-18

**Authors:** Febitha Kandan-Kulangara, Rashmi G. Shah, El Bachir Affar, Girish M. Shah

**Affiliations:** 1 Laboratory for Skin Cancer Research, Department of Molecular Biology, Medical Biochemistry and Pathology, Faculty of Medicine, Hospital Research Centre of Laval University (CHUL/CHUQ), Laval University, Quebec, Quebec, Canada; 2 Department of Medicine, Faculty of Medicine, Maisonneuve Rosemont Hospital Research Center, Montreal University, Montreal, Quebec, Canada; Universität Heidelberg, Germany

## Abstract

Gene silencing by transient or stable RNA-interference (RNAi) is used for the study of apoptosis with an assumption that apoptotic events will not influence RNAi. However, we recently reported that stable RNAi, i.e., a permanent gene-knockdown mediated by shRNA-generating DNA vectors that are integrated in the genome, fails rapidly after induction of apoptosis due to caspase-3-mediated cleavage and inactivation of the endoribonuclease Dicer-1 that is required for conversion of shRNA to siRNA. Since apoptosis studies also increasingly employ transient RNAi models in which apoptosis is induced immediately after a gene is temporarily knocked down within a few days of transfection with RNAi-inducing agents, we examined the impact of apoptosis on various models of transient RNAi. We report here that unlike the stable RNAi, all forms of transient RNAi, whether Dicer-1-independent (by 21mer dsRNA) or Dicer-1-dependent (by 27mer dsRNA or shRNA-generating DNA vector), whether for an exogenous gene GFP or an endogenous gene poly(ADP-ribose) polymerase-1, do not fail for 2–3 days after onset of apoptosis. Our results reflect the differences in dynamics of achieving and maintaining RNAi during the early phase after transfection in the transient RNAi model and the late steady-state phase of gene-knockdown in stable RNAi model. Our results also sound a cautionary note that RNAi status should be frequently validated in the studies involving apoptosis and that while stable RNAi can be safely used for the study of early apoptotic events, transient RNAi is more suitable for the study of both early and late apoptotic events.

## Introduction

RNA-interference (RNAi) is a mechanism for sequence-specific silencing of a gene by 21–23mer dsRNA, also called small interfering RNA (siRNA) which guides RNA-induced silencing complex (RISC) containing the endoribonuclease of the Argonaut family (Ago) to search and destroy the target mRNA [1], [2]. In mammalian cells, transient RNAi, i.e., knockdown of a target gene for a few days can be achieved rapidly after transfection with a synthetic 21mer dsRNA or its precursors, such as 27mer dsRNA [3] or a short hairpin RNA (shRNA)-generating DNA vector [4]. While the transfected 21mer dsRNA/siRNA is directly incorporated in the RISC, the 27mer dsRNA or DNA vector-derived shRNA need to be converted first to siRNA by the endoribonuclease Dicer-1. In transient RNAi models, the gene expression returns to normal once siRNA or its precursors are degraded; and the siRNA-loaded RISC molecules are depleted due to dilution with cell division or metabolic instability in the absence of target mRNA [1]. Stable RNAi, on the other hand, can be achieved when shRNA-generating DNA vector is integrated in the genome under selection pressure, so that its transcription results in a continuous supply of shRNA molecules and stable knockdown of the target gene [4]. Both transient and stable RNAi are being exploited in mammalian cells for examining various cellular processes [2], and more specifically to study apoptosis with an assumption that these RNAi processes would not be affected by apoptosis. However, recently we reported that stable RNAi fails soon after induction of apoptosis because of caspase-mediated cleavage and inactivation of Dicer-1, which is required to form siRNA from DNA vector-derived shRNA [5]. However, the impact of apoptosis on transient RNAi has never been examined although some apoptosis studies use Dicer-1-dependent transient RNAi achieved with 27mer dsRNA [6] or the shRNA-generating DNA vectors [7]. Hence, we characterized apoptotic fate of Dicer-1-dependent and independent forms of transient RNAi of an exogenous and an endogenous gene and compared it with stable RNAi. We report here that while Dicer-1-dependent stable RNAi rapidly fails after onset of apoptosis, the transient RNAi, whether dependent on Dicer-1 or not, continues to knockdown the target genes for several days after onset of apoptosis, reflecting the differences in dynamics of achieving RNAi in transient and stable RNAi.

## Results

### Persistence of transient RNAi whereas failure of stable RNAi of stably expressed GFP

**Figure 1:**
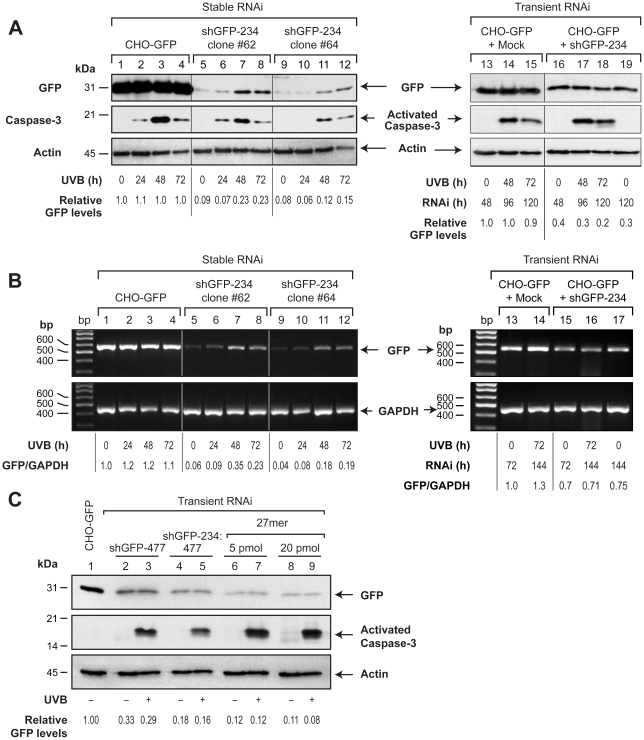
Failure of stable but not transient RNAi of stably expressed GFP during apoptosis in CHO-GFP cells. (**A**) Comparison of GFP protein knockdown levels during stable versus transient RNAi by shGFP-234. For stable RNAi (left panel), CHO-GFP parental cells and its two stable shGFP-234 clones #62 and 64 were treated with 1,600 J/m^2^ UVB and samples were harvested from 24–72 h for immunoblotting. For transient RNAi (right panel), CHO-GFP cells were transfected with shGFP-234 (or mock pCMV vector) for 48 h, treated with UVB and harvested from 48–72 h for immunoblotting. Caspase-3 probing served to confirm apoptosis and actin-probing served as a loading control. GFP-levels are expressed relative to the untreated CHO-GFP controls (lanes 1 and 13). (**B**) Comparison of GFP transcript levels during stable versus transient RNAi by shGFP-234. From the cells treated as above, total RNA was extracted and subjected to RT-PCR determination of mRNA for GFP and GAPDH, the ratio of GFP/GAPDH is expressed as relative to respective untreated control (lanes 1 and 13). For both A and B panels, experiments were repeated 3–4 times with identical results. (**C**) Comparison of GFP protein knockdown levels in cells with variable extent of transient RNAi introduced by shGFP-234, shGFP-477 or 27mer dsRNA. The CHO-GFP cells were transfected with 3 µg shGFP-477 alone or in (1∶2) combination with shGFP-234, and with different concentrations of 27mer dsRNA. The control CHO-GFP cells were mock transfected with unrelated shRNA-generating DNA vector. At 72 h after transfection, unirradiated cells were harvested (UVB-) and the rest were treated with 1,600 J/m^2^ UVB and harvested after 72 h (UVB+). The samples were probed for GFP and activated caspase-3, whereas actin probing served as a loading control. GFP-levels are expressed relative to the untreated CHO-GFP controls (lane 1). The experiments were repeated 4 times with identical results.

We first compared the apoptotic fate of transient and stable RNAi of GFP which were achieved using the same shRNA-generating DNA vector shGFP-234 in the cells that constitutively express high levels of GFP (CHO-GFP) (**Fig. 1**). For stable RNAi, CHO-GFP cells were transfected with shGFP-234 and clones with permanent knockdown of GFP were isolated over several weeks after transfection. A semi-quantitative analyses of GFP signals revealed that two of these shGFP-234 clones #62 and #64 had stable and significant (>90%) knockdown of GFP, when compared to GFP expression in the control CHO-GFP cells (**Fig. 1A**, lanes 1, 5 and 9). For transient RNAi, CHO-GFP cells were transfected with shGFP-234 for 48 h to obtain ∼60% knockdown of GFP (**Fig. 1A**, lanes 13 and 16). Apoptosis was induced in both the RNAi models by treatment with ultraviolet B (UVB) and the fate of RNAi was monitored for further 72 h. In the CHO-GFP cells, high levels of GFP expression present prior to induction of apoptosis remained unchanged up to 72 h after UVB-treatment that caused formation of activated caspase-3 (**Fig. 1A**, lanes 1–4). Thus, GFP gene expression or protein stability *per se* are not altered during apoptosis. In the two stable shGFP clones where the expression of GFP was significantly knocked down prior to apoptosis, UVB-induced apoptosis resulted in a failure of RNAi, i.e., an increase in GFP levels by 48–72 h concomitant with caspase-3 activation (**Fig. 1A**, lanes 5–12). This is in agreement with our earlier demonstration of apoptosis-associated failure of stable RNAi for three other genes, namely poly(ADP-ribose) polymerase-1 (PARP), p14^ARF^ and lamin A/B [5]. Interestingly, transient RNAi by the same shGFP-234, which was achieved prior to induction of apoptosis, did not fail for up to 72 h after exposure to UVB, because the GFP signal did not increase in the 120 h-UVB-treated cells as compared to 120 h-untreated cells (**Fig. 1A**, lanes 16-19).

To determine whether changes in GFP protein levels were indeed due to the influence of RNAi on the availability of GFP transcripts, the relative abundance of mRNA for GFP over control gene GAPDH were analyzed in these two models by RT-PCR (**Fig. 1B**). In the CHO-GFP cells without any RNAi, the GFP transcript levels before and after induction of apoptosis were not significantly altered confirming that apoptosis does not directly influence transcription of GFP or the stability of its mRNA (**Fig. 1B**, lanes 1-4). In two stable RNAi clones, the relative GFP mRNA levels were significantly (4-6× fold) upregulated after induction of apoptosis as compared to the levels seen prior to apoptosis (**Fig. 1B** **,** lanes 5-8 and 9-12), indicating failure of stable RNAi of GFP and the accumulation of its transcripts from 24 h onwards. In contrast, in the transient RNAi model, a relatively moderate decrease in GFP transcripts (30%) at 72 h after transfection and prior to induction of apoptosis (**Fig. 1B** **,** lanes 13 and 15) was not significantly reversed for up to 72 h after UVB-induced apoptosis (**Fig. 1B** **,** lanes 15 and 16), indicating the persistence of transient RNAi during apoptosis. Thus, despite originating from the same shGFP-234 DNA vector, the stable RNAi of GFP rapidly failed by 24–48 h, whereas the transient RNAi persisted up to 3 days after onset of apoptosis.

It is plausible that apoptosis-associated failure of RNAi did not become evident in the above described transient RNAi model simply because it started with a much lesser extent of knockdown of GFP (60%) as compared to highly efficient (91–92%) knockdown seen in stable RNAi model. To address this issue, we employed a variety of transient transfection conditions, such as use of a different shRNA-generating DNA vector (shGFP-477), a combination of two shGFP DNA vectors or a 27mer dsRNA to achieve much greater extent of transient GFP knockdown (**Fig. 1C**). The DNA vector shGFP-477 or a combination of shGFP-234 and shGFP-477, yielded 67 and 82% GFP-knockdown, respectively at 72 h after transfection (**Fig. 1C**, lanes 1, 2 and 4). An even greater extent of knockdown (88–89%) was achieved at 72 h after transient transfection with 5 or 20 pmol of 27mer dsRNA (**Fig. 1C**, lanes 1, 6 and 8). In each of these transient RNAi models, the signal for GFP did not increase for up to 72 h after UVB-treatment that caused formation of activated caspase-3 (**Fig. 1C**, lanes 2–9: compare lanes +/− UVB in each set), confirming that irrespective of the extent of knockdown from 60–89%, transient RNAi did not fail for up to 3 days after onset of apoptosis.

### Persistence of GFP-knockdown during apoptosis in stable RNAi clones supplemented with transient RNAi

**Figure 2:**
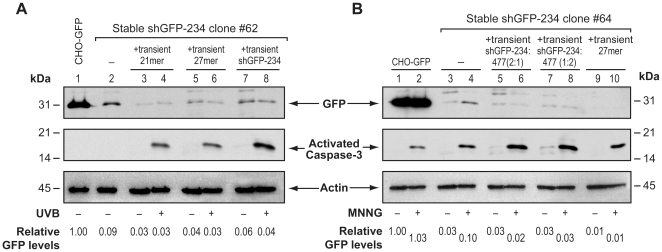
Persistence of GFP-knockdown during apoptosis in stable RNAi cells co-expressing transient RNAi. (**A**) Effect of UVB-induced apoptosis on RNAi of GFP in the stable RNAi clone #62 supplemented with transient RNAi. The shGFP-234 clone #62 with a stable RNAi of constitutively expressed GFP was transiently transfected with one of the three GFP-targeting RNAi-inducing agents: 120 pmol of 21mer dsRNA, 2 pmol of 27mer dsRNA or 3 µg of shGFP-234 or with 3 µg of unrelated shRNA–generating DNA vector as control (mock) for transfection. At 48 h after transfection, unirradiated cells were harvested (UVB-) and the rest were treated with 1,600 J/m^2^ UVB (UVB+) and harvested after 72 h. (**B**) Effect of MNNG-induced apoptosis on RNAi of GFP in the stable RNAi clone #64 supplemented with transient RNAi. The shGFP-234 clone #64 was transiently transfected with 3 µg of shGFP-234 and shGFP-477 DNA vector (2∶1 and 1∶2) in combination or with 5 pmol of 27mer dsRNA for 48 h. Mock control was transfected as mentioned above. At 48 h after transfection, untreated cells were harvested (MNNG-), while the rest were treated with 300 µM MNNG (MNNG+) and harvested 24 h later. For both the panels, the samples were probed for GFP and activated caspase-3, whereas actin probing served as a loading control. The experiments were repeated 4 times with identical results.

As above studies examined transient and stable RNAi in different cellular models, we next examined the apoptotic fate of GFP-knockdown in the cells containing both stable and transient RNAi (**Fig. 2**). To the shGFP-234 clone #62 that has a pre-existing (91%) stable RNAi of GFP (**Fig. 2A**, lanes 1 and 2), we added transient RNAi by transfection with GFP-targeting 21mer dsRNA, 27mer dsRNA or shGFP-234 DNA vector, which resulted in a total knockdown of 94–97% at 48 h after transfection (**Fig. 2A**, lanes 1, 2, 3, 5 and 7). When apoptosis was induced by UVB in these cells, it resulted in formation of activated caspase-3, but there was no increase in GFP signal at 72 h after UVB treatment (**Fig. 2A**, lanes 3–8: paired lanes +/− UVB). Thus, although stable RNAi failed during apoptosis (**Fig. 1A**), addition of transient RNAi allowed the same cells to continue to knockdown GFP during apoptosis. To confirm the general nature of above observation, we used another stable clone #64 for shGFP-234 and induced apoptosis with *N*-methyl-*N'*-nitroso-*N*-nitrosoguanidine (MNNG) instead of UVB (**Fig. 2B**). In this clone, the original 97% stable GFP-knockdown (**Fig. 2B**, lanes 1 and 3) was slightly improved (from <1 to 2%) after addition of transient RNAi with two different combinations of shGFP-234 and shGFP-477 DNA vectors or 27mer dsRNA (**Fig. 2B**, lanes 1,3,5,7 and 9). When apoptosis was induced by MNNG in these cells, the GFP signal increased by 3× fold in the cells which had only stable RNAi (**Fig. 2B**, lanes 3 and 4), confirming apoptosis-associated abrogation of stable RNAi. However, GFP signal did not increase in the cells which had transient RNAi added to the stable RNAi (**Fig. 2B**, lanes 5–10, compare lanes +/− MNNG), confirming that GFP knockdown by transient RNAi continued to remain functional in a background where stable RNAi was failing. We also confirmed in the stable clone #64 with or without added transient RNAi by shGFP-234 that UVB-induced apoptosis caused a failure of RNAi in cells with stable RNAi alone but not in the cells with stable plus transient RNAi (Supplementary Figure S1). Thus, even in the same cell, while stable RNAi of GFP failed, addition of transient RNAi allowed continued knockdown of GFP during apoptosis, confirming persistence of transient RNAi during UVB or MNNG-induced apoptosis.

### Persistence of different forms of transient RNAi of co-expressed GFP during apoptosis

**Figure 3:**
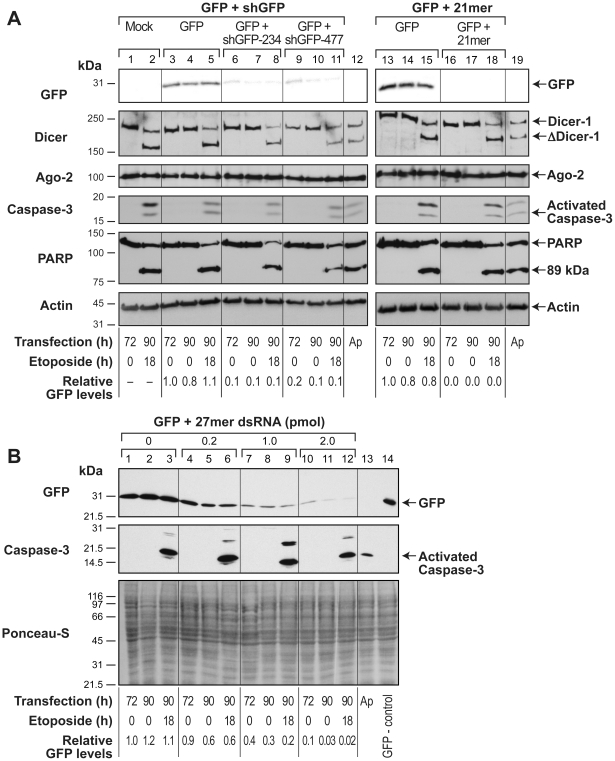
Persistence of transient RNAi of co-transfected GFP during apoptosis in GM637 cells. For examining apoptotic fate of transient RNAi of co-expressed GFP, GM637 cells were co-transfected with a GFP-expression vector and one of the following RNAi-inducing agents: (**A**) shGFP-234, shGFP-477 and 21mer dsRNA or mock pCMV vector; or (**B**) Varying amounts (0, 0.2, 1.0 and 2.0 pmol) of 27mer dsRNA. At 72 h of knockdown, apoptosis was induced by treatment with 100 µM etoposide (or mock-control) for 18 h. Samples were harvested at 72 h (before apoptosis) or 90 h (after apoptosis) for immunoblotting for GFP, Dicer-1, Ago-2, activated caspase-3 and PARP, as specified for each panel. The relative GFP values were determined as described in Fig. 1A. For above panels, either Ponceau-S staining or actin immunoprobing served as loading controls and Ap refers to apoptotic cell extract prepared from etoposide-treated HL-60 cells. The experiments were repeated at least four times with identical results.

While above studies examined RNAi of target gene GFP that is stably expressed, many transient RNAi studies examine knockdown of a co-transfected target gene; hence we examined apoptotic fate of transient RNAi of a co-transfected GFP. In the human skin fibroblasts, GFP-expression vector was co-transfected with two different shGFP DNA vectors (#234 and 477) or a 21mer dsRNA. The GFP expression at 72 h after transfection of the GFP-expression vector alone was significantly suppressed (80–99%) when co-transfected with all three transient RNAi-inducing agents (**Fig. 3A**, 72 h lanes). At this stage, when apoptosis was induced by treatment with etoposide for 18 h, it did not result in any increase in GFP levels at 90 h (i.e., 72 h transfection plus 18 h etoposide) over its respective 90 h non-apoptotic control in each of the RNAi model (**Fig. 3A**, paired 90 h lanes +/− etoposide), confirming the persistence of Dicer-1-dependent and independent mode of transient RNAi of co-transfected GFP during apoptosis.

Further analyses of these cells revealed that etoposide-treatment caused activation of caspase-3 and cleavage of PARP to its 89 kDa fragment, accompanied by apoptosis-specific cleavage of Dicer-1 to a 180 kDa fragment [5], whereas RISC-associated Ago-2 remained intact (**Fig. 3A**, lanes 2, 5, 8, 11, 15 and 18). The presence of intact Ago-2 during apoptosis was in agreement with the persistence of RISC-mediated transient RNAi by 21mer dsRNA. However, despite the cleavage of Dicer-1 to its 180 kDa fragment, which results in its catalytic inactivation [5], pre-existing transient RNAi by the shRNA-generating DNA vector did not fail for at least 18 h after induction of apoptosis.

To examine whether the extent of gene knockdown plays a role in the persistence of transient RNAi during cell death, we obtained variable extent of GFP-knockdown with increasing amounts of Dicer-1-substrate 27mer dsRNA. The co-transfection of GFP-expression plasmid with 0.2, 1 or 2 pmol of 27mer dsRNA caused 10, 60 or 90% GFP-knockdown, respectively at 72 h (**Fig. 3B**, lanes 1, 4, 7 and 10). The induction of apoptosis with etoposide treatment resulted in activation of caspase-3 but caused no significant change in GFP at all three levels of GFP-knockdown (**Fig. 3B**, paired 90 h lanes +/− etoposide), indicating that persistence of Dicer-1-dependent transient RNAi during apoptosis was not influenced by the extent of gene-knockdown.

### Persistence of different forms of transient RNAi of PARP during apoptosis

**Figure 4:**
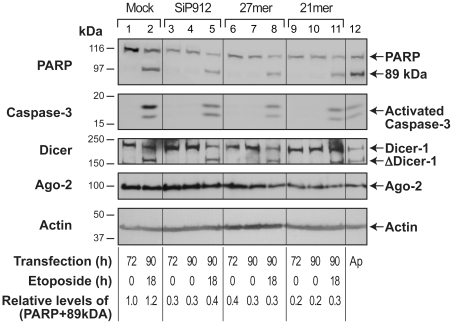
Persistence of transient RNAi of endogenous gene PARP during apoptosis in GM637 cells. The GM637 cells were transiently transfected with three RNAi-inducing agents: shRNA-generating DNA vector-SiP912, 27mer dsRNA or 21mer dsRNA or mock pCMV vector as control. At 72 h after transfection, cells were treated with 100 µM etoposide or with solvent control for 18 h and samples were harvested for immunoblotting for PARP, activated caspase-3, Dicer-1 and Ago-2. The actin probing served as loading controls and Ap refers to apoptotic cell extract prepared from etoposide-treated HL-60 cells. The (PARP+89-kDa) signals from each sample are expressed as relative to untreated control in lane 1. The results shown in all three above panels are representative of 3–5 independent experiments with identical results.

Finally, since GFP is an exogenous gene, we examined whether transient RNAi of an endogenous and constitutively expressed gene PARP will also persist during apoptosis (**Fig. 4**). In the human skin fibroblasts, transient RNAi of PARP with shRNA-generating DNA vector SiP912 [8], 27mer dsRNA or 21mer dsRNA for 72 h resulted in 60–80% PARP-knockdown as compared to PARP-levels seen in the cells without RNAi (**Fig. 4**, 72 h lanes). Induction of apoptosis by 18 h treatment with etoposide at this stage resulted in activation of caspase-3 and cleavage of the residual PARP to its 89 kDa fragment, but no net increase in the total amount of PARP and its 89 kDa fragment after apoptosis in all models of transient RNAi (**Fig. 4** **,** 90 h lanes +/− etoposide). Etoposide treatment consistently induced cleavage of Dicer-1 to its 180-kDa fragment (**Fig. 4**, 90 h lanes), indicating that despite cleavage of Dicer-1, transient RNAi of the endogenous gene PARP by Dicer-1-independent (21mer dsRNA) or Dicer-1-dependent (shRNA and 27mer dsRNA) mechanisms persisted during apoptosis.

## Discussion

We show here that transient RNAi of endogenous or exogenous gene, which has been achieved after short-term transfection with the gene-targeting 21mer dsRNA (siRNA), 27mer dsRNA or shRNA-generating DNA vector, does not fail for 2–3 days after onset of apoptosis. The results with 21mer dsRNA and detection of intact Ago-2 during apoptosis confirm our earlier report that RISC/Ago-2-mediated transient RNAi continues to function normally during apoptosis [5]. However, our results showing the persistence of transient RNAi by Dicer-1-dependent 27mer dsRNA or shRNA for up to 2–3 days during apoptosis is in contrast to the rapid failure of shRNA-mediated stable RNAi, which was reported earlier [5] and confirmed here. Since apoptotic cleavage of Dicer-1 to 180 kDa fragment results in its catalytic inactivation [5], it is evident that the production of new siRNA molecules from its precursors by the action of Dicer-1 would be halted with the onset of apoptosis in both the models of RNAi. However, this seems to interrupt only the stable RNAi and not the transient RNAi, indicating that the dynamics of achieving gene-knockdown and the dependency on Dicer-1 function must be different in stable and transient RNAi.

At least four factors could be responsible for the difference in the response of transient versus stable RNAi during apoptosis: (i) stability of siRNA-loaded RISC molecules; (ii) availability of siRNA in the cells at the time of onset of apoptosis; (iii) the half-life of siRNA; and (iv) the optimum number of siRNA-loaded RISC molecules required for an efficient gene knockdown. It has been suggested that siRNA-loaded RISC may be very stable [1], which could readily explain the persistence of all forms of transient RNAi during apoptosis, because once formed prior to onset of apoptosis, the siRNA-loaded RISC molecules could continue to knockdown the gene for several more days or until the cell dies by apoptosis. However, this does not explain why stable RNAi which also has an efficient (>90%) gene knockdown by siRNA-loaded RISC prior to induction of apoptosis, fails after onset of apoptosis. Our results with stable RNAi strongly indicate that siRNA-loaded RISC cannot be very stable, and that input of siRNA at regular intervals is required for the RISC to continue to knockdown a gene; thus making it potentially susceptible to interruption in the supply of siRNA due to cleavage/inactivation of Dicer-1. Thus, an RNAi model in which the cells have higher supply of siRNA at the time of onset of apoptosis is likely to persist for a few days during apoptosis, whereas one with a minimal steady-state supply of siRNA is likely to be abrogated rapidly due to rapid exhaustion of its siRNA. In the transient RNAi, since there is a massive influx of siRNA or its precursor (27mer dsRNA or shRNA-generating DNA), a large presence of preformed or Dicer-1-generated siRNA can be expected to remain in the cells for a few more days during which apoptosis is induced in this model. In contrast, the stable RNAi clones are isolated over several weeks after initial transfection; hence these cells would have lost all of the initially transfected free shRNA-generating DNA molecules. Hence, stable RNAi would become totally dependent on the steady-state synthesis of shRNA from the shRNA-generating DNA integrated in the genome.

The second most important issue after availability of siRNA at the onset of apoptosis would be the half-life of the siRNA in cells. In blood, siRNA has been reported to have half-life of few minutes [1], however a recent study showed that siRNA can have a considerably longer half-life of up to 96 h in some cells [9]. Our results showing persistence of transient RNAi for several days after failure of Dicer-1 supports the argument that siRNA available at the onset of apoptosis must have sufficiently long half-life to persist for several days and support the RISC-mediated knockdown of the gene. In contrast, the stable RNAi clones appear to be rapidly exhausted of the siRNA, indicating a much lower steady-state levels of siRNA. Our data that additional transient RNAi can be introduced in the clones with stable RNAi (**Fig. 2**) supports the argument that stable RNAi clones do not produce saturating amounts of siRNA to load all the available the RISC molecules. In addition, based on microRNA data, it has been estimated that about 1000 siRNA molecules per cell can efficiently silence a gene [1], and this number of siRNA would be readily available for several days after onset of apoptosis in the transient RNAi model as stated above. In contrast, since siRNA and miRNA share the same RISC machinery in mammalian cells, a thriving stable RNAi clone would make just the sufficient quantity of shRNA to efficiently silence the gene while leaving the remaining RISC molecules available for important cellular functions by miRNA. Thus stable RNAi, operating with just the minimally required number of siRNA-loaded RISC molecules, would be more prone than transient RNAi to the apoptotic inactivation of Dicer-1 and interruption in the supply of fresh siRNA.

In conclusion, our results sound a cautionary note that while using RNAi in the study of apoptosis, one must monitor and account for a possible failure of RNAi after onset of apoptosis. Our results validate the use of stable RNAi mainly for the study of early apoptotic events and transient RNAi for study of both early and late apoptotic events. Our results indicate that siRNA loaded RISC is not very stable because it needs frequent input of fresh siRNA and cellular siRNA formed or introduced in the cells during transient RNAi is more stable than in blood. Thus 21mer and 27mer siRNA have a good therapeutic potential provided their stability could be increased in blood, because once inside the cells, they can achieve knockdown of the target gene for a long period of time.

## Materials and Methods

### Cells

The human diploid SV-40 transformed skin fibroblast cells GM00637 (Coriell Cell Repository, Camden, NJ) and hamster cell line CHO (sub-line WT-5) were cultured in αMEM (Gibco) supplemented with 10% foetal bovine serum, 50 U/ml penicillin and 50 µg/ml streptomycin at 37°C in a humidified incubator with 5% CO_2_.

### Creation of CHO-derived clones with stable expression of GFP (CHO-GFP) and stable shRNA-mediated RNAi of GFP (CHO-shGFP)

The details of creation of CHO-GFP clones and its stable RNAi clones are provided in Methods S1. In brief, CHO cells were transfected with pEGFP-N1 (Clontech) and strong GFP-expressing clones were selected, one of which was used in the present study. For stable RNAi of GFP, CHO-GFP clone was transfected with pBS-U6-based [8] and shRNA-generating DNA vector shGFP-234 along with pTK-Hyg plasmid to isolate stable hygromycin-resistant shGFP clones, two of which were used in the study. The extent of knockdown of GFP was measured relative to the GFP levels expressed in untreated CHO-GFP control cells.

### Transient RNAi of GFP or PARP

The details of all transient transfection conditions are provided in Methods S1. In brief, for the transient RNAi of constitutively expressed GFP, CHO-GFP cells were transfected with GFP-targeting DNA vectors shGFP-234, shGFP-477 or 27mer dsRNA for 48–72 h prior to induction of apoptosis. For introduction of transient RNAi in the cells with stable RNAi of GFP, the shGFP-234-clone #62 or clone #64 were transfected with GFP-targeting 21mer dsRNA, 27mer dsRNA, shGFP-234 or shGFP-477 DNA vector for 48 h prior to induction of apoptosis. The mock control cells were transfected with equivalent amount of blank vector pCMV DNA or unrelated shRNA-generating DNA vector. For transient RNAi of co-expressed GFP, GM637 cells were transfected with pEGFP-N1 (Clontech) cDNA or CMV plasmid (as mock DNA) with or without GFP-targeting 21mer dsRNA, 27mer dsRNA, shGFP-234 or shGFP-477. For transient RNAi of PARP, GM637 cells were transfected with one of the following PARP-targeting 21mer dsRNA, 27mer dsRNA (IDT) or shRNA-directing SiP912 DNA vector [8].

### Apoptosis-inducing treatments and Western blotting

Apoptosis was induced in different models with either 1,600 J/m^2^ UVB (Spectrolinker XL-1000 UV cross-linker), 300 µM MNNG or 100 µM etoposide for specified time. The cell extracts were prepared and samples representing 200,000 cells or 10–20 µg protein were resolved on 6, 8 or 12% SDS-PAGE, blotted on nitrocellulose and probed with following antibodies: GFP (Roche, 1∶5,000); PARP (C-2-10, Aparptosis Inc., 1∶10,000); human Dicer-1 (Abcam, 1∶1,000); human Ago-2 (Wako, 1∶200); activated caspase-3 (Cell Signaling, 1∶1,000) and Actin (Sigma, 1∶20,000). The immunoblots were either read directly for chemiluminescence or autoradiographic films were scanned using ChemiGenius 2 Bio Imaging system (SynGene) and quantification of signals was carried out using GeneTools software version 4.00 (SynGene).

### Extraction of total RNA and RT-PCR

The details of RNA preparation, RT-PCR primers and cycle conditions as well as data analyses are provided in Methods S1. In brief, total RNA was prepared from 1–3×10^6^ cells using mirVana™ PARIS™ kit (Ambion). The RT-PCR was carried out using OneStep RT-PCR kit (Qiagen) and the MasterCycler Personal PCR-machine (Eppendorf Life Science). The GFP or GAPDH bands after resolution on 1.5% agarose gel were quantified using ChemiGenius 2 with GeneTools software (SynGene).

## Supporting Information

Figure S1 Abrogation of stable RNAi but persistence of transient RNAi in same cells during UVB-induced apoptosis. The shGFP-234 clone #64 was transiently transfected with 3 µg of shGFP-234 DNA vector for 48 h. The CHO-GFP parental cells and shGFP-234 cells with or without additional transient RNAi by shGFP-234 or unrelated shRNA-generating DNA vector (control) were treated either with 1,600 J/m^2^ UVB or mock-irradiated. The samples were harvested at 72 h and probed for GFP and activated caspase-3, whereas actin probing served as a loading control. The experiments were repeated 3 times with identical results. GFP-levels are expressed relative to the untreated CHO-GFP controls (lane1).
(0.56 MB TIF)

Methods S1 Detailed methods for creation of clones, transfections and RT-PCR.
(0.04 MB DOC)
