# Supplementary figures and images for: Persistence of Different Forms of Transient RNAi during Apoptosis in Mammalian Cells
Source: PLoS One. 2010 Aug 18;5(8):e12263. doi: 10.1371/journal.pone.0012263 (PMC2923616; doi:10.1371/journal.pone.0012263)

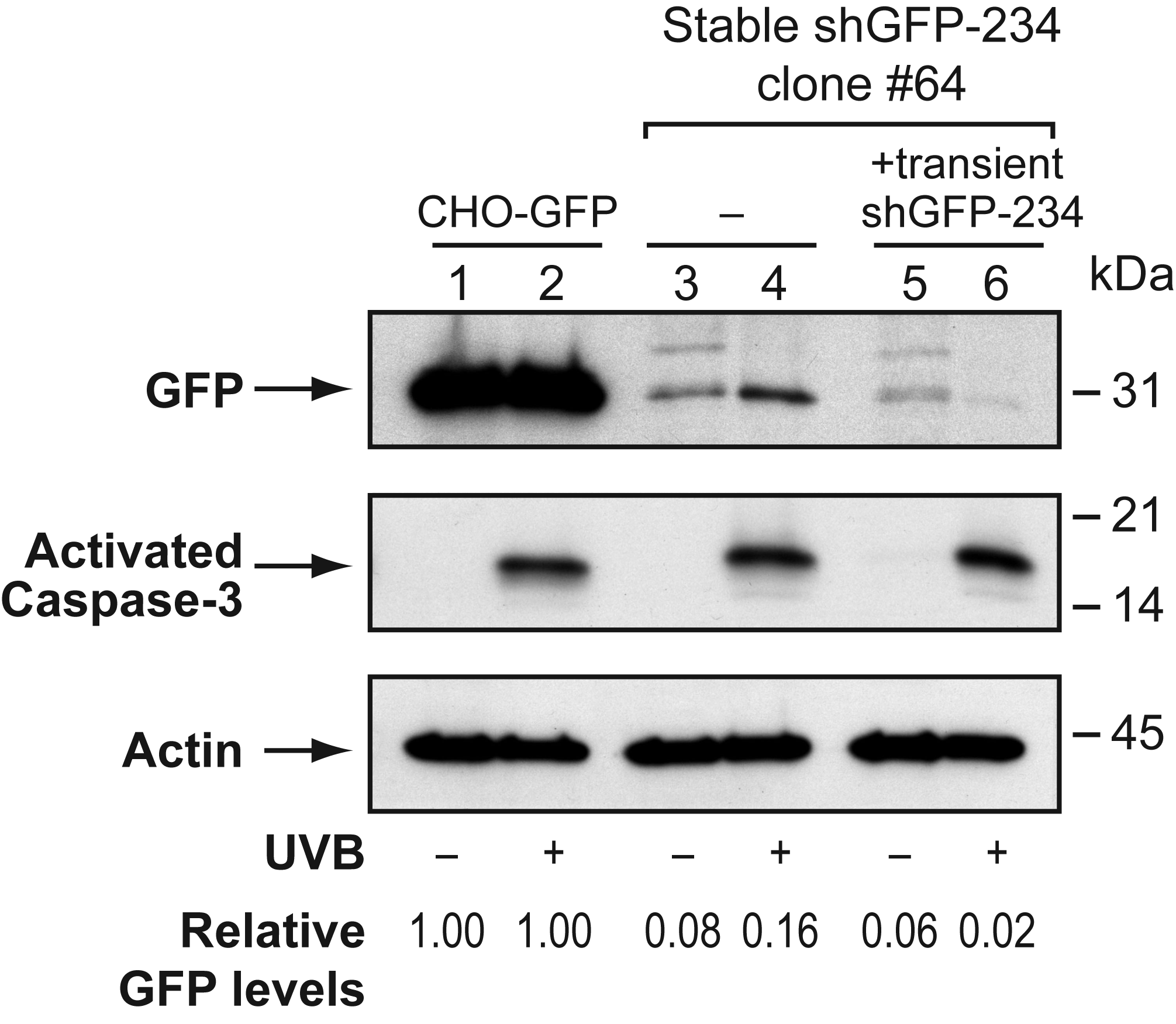

Supplement: Figure S1 — Abrogation of stable RNAi but persistence of transient RNAi in same cells during UVB-induced apoptosis. The shGFP-234 clone #64 was transiently transfected with 3 µg of shGFP-234 DNA vector for 48 h. The CHO-GFP parental cells and shGFP-234 cells with or without additional transient RNAi by shGFP-234 or unrelated shRNA-generating DNA vector (control) were treated either with 1,600 J/m2 UVB or mock-irradiated. The samples were harvested at 72 h and probed for GFP and activated caspase-3, whereas actin probing served as a loading control. The experiments were repeated 3 times with identical results. GFP-levels are expressed relative to the untreated CHO-GFP controls (lane1). (0.56 MB TIF) [file pone.0012263.s001.tif]
