# Supplementary material for: Persistence of Different Forms of Transient RNAi during Apoptosis in Mammalian Cells
Source: PLoS One. 2010 Aug 18;5(8):e12263. doi: 10.1371/journal.pone.0012263 (PMC2923616; doi:10.1371/journal.pone.0012263)
Supplement: Methods S1 — Detailed methods for creation of clones, transfections and RT-PCR. (0.04 MB DOC) [file pone.0012263.s002.doc]

**Supplementary Material: Detailed Methods**

**Creation of CHO-derived cells with stable expression of GFP (CHO-GFP).** The CHO cells with constitutive expression of GFP (CHO-GFP cells) were created by stable transfection of 80% confluent cells in a 60 mm dish with 3 µg pEGFP-N1 (Clontech) using Lipofectamine 2000 (Invitrogen), as per the manufacturer’s protocol. The GFP-expressing clones were selected and maintained in a medium containing 800 and 400 µg/ml geneticin, respectively. One of these CHO-GFP clones was used in the study.

**Creation of clones with stable shRNA/DNA vector-based RNAi of GFP (CHO-shGFP).** For stable RNAi of GFP, CHO-GFP clone was transfected with pBS-U6-based (Shah et al, 2005) and shRNA-generating DNA vector shGFP-234 containing GFP targeting sequence 5’-GCA TGA AGC AGC ACG ACT TCT T-3’ along with pTK-Hyg plasmid to isolate stable hygromycin-resistant shGFP clones, which were maintained in the medium containing 400 µg/ml geneticin and 200 µg/ml hygromycin. Two of these shGFP-234 clones #62 and 64 were used in the study.

**Transient RNAi of GFP or PARP.** For transient RNAi of constitutively expressed GFP, CHO-GFP cells at 75-80% confluence in 60 mm dishes were transfected with either Lipofectamine-2000 or CaPO_4_ protocols with specified amounts of shRNA-generating DNA vectors or dsRNA of 27mer or 21mer. After 24h, cells from each dish were trypsinized and distributed in multiple 60 mm dishes to be used for control or apoptotic treatments. At 48 or 72h after transfection, apoptosis-inducing treatments were carried out as described below.

For transient RNAi of GFP in the cells having stable RNAi of GFP, the CHO-shGFP-234 clone was transfected as above with one of the following GFP-RNAi-inducing agents: 120 pmol of 21mer dsRNA based on the sequence 5’-GCA AGC UGA CCC UGA AGU UCA U-3’ (Qiagen); 2 or 5 pmol of 27mer dsRNA based on the sequence 5’-ACC CUG AAG UUC AUC UGC ACC ACC G-3’ (Qiagen); or 3 µg DNA vectors shGFP-234 or shGFP-477 DNA vector based on the sequence 5’-GAA CGG CAT CAA GGT GAA CTT-3’. After transfection, cells were processed as mentioned above.

For transient RNAi of co-expressed GFP, GM637 cells, under culture and transfection conditions described above, were transfected with 0.2 µg of pEGFP-N1 (Clontech) cDNA or CMV plasmid (as mock DNA) with 120 pmol of 21mer dsRNA, 0.2, 1 and 2 pmol of 27mer dsRNA or 1 µg of shGFP-234 or with shGFP-477. After transfection, cells were processed as mentioned above.

For transient RNAi of PARP, GM637 cells were transfected under conditions described above except that cellular PARP was targeted for knockdown with one of the following PARP-RNAi-inducing agents: 600 pmol of 21mer (IDT); 600 pmol of 27mer dsRNA (IDT) or 5 µg shRNA-directing SiP912 DNA vector (Shah et al, 2005). The PARP-targeting sequence in each of the dsRNA was based on the sequence described earlier for the SiP912 (Shah et al, 2005). After transfection, cells were processed as mentioned above.

**Extraction of total RNA and RT-PCR:** Total RNA from cells was extracted using the mirVana^TM^ PARIS^TM^ kit (Ambion). In brief, 1-3 x 10^6^ cells were trypsinized, washed with PBS and the pellet was extracted using 300-600 µl of disruption buffer provided with the kit. The lysate was mixed with equal volume of 2X denaturation buffer, extracted with acid phenol and the RNA was obtained from the aqueous phase, using the filter cartridge provided in the kit. Elution was performed with 50 µl RNase-free water and RNA was estimated spectrophotometrically. The quality of RNA was confirmed by loading 1 µg on formaldehyde-agarose gel and RNA was stored at -80°C.

The RT-PCR was carried out using the OneStep RT-PCR kit (Qiagen) and the MasterCycler Personal PCR-machine (Eppendorf Life Science). The reaction components were as per the instructions for the one-step PCR, without the use of RNase-inhibitor or the Q solution. The following primer-set for GFP resulted in a DNA product of 530 bp: Forward GFP primer: 5’ CAA GCT GAC CCT GAA GTT CAT C 3’; Reverse GFP primer: 5’ GAA CTC CAG CAG GAC CAT GTG 3’. The following primer-set for GAPDH resulted in a DNA product of 473 bp: Forward GAPDH primer: 5’ CTT CAT TGA CCT CAA CTA CAT GG 3’; Reverse GAPDH primer: 5’ GTC TTC TGG GTG GCA GTG ATG 3’. The reaction was performed in a total volume of 25 µl containing 0.6 µM of each of the two primers of the set and 1 µg total RNA. The thermal cycler was set at 50°C before transferring the tubes containing the reaction mixture from ice and the reaction was performed for 25 cycles. At the end of the reaction, 7.5 µl of the GFP or GAPDH mixture was separated on 1.5 % agarose gel in 1X TBE for one hour at 100V. The bands stained with ethidium bromide were viewed on a transilluminator using ChemiGenius 2 Bio Imaging system (SynGene) and quantification of signals was carried out using GeneTools software version 4.00 (SynGene).

**REFERENCE**

Shah RG, Ghodgaonkar MM, Affar EB, Shah GM (2005) DNA vector-based RNAi approach for stable depletion of poly(ADP-ribose) polymerase-1*. Biochem Biophys Res Comm*u**n 3**31(**1**): 167-174
